# Zero-Shot Domain Adaptation of 2D Mammography Lesion Detection Model for 3D Breast CT Malignancy Detection

**DOI:** 10.3390/cancers18162585

**Published:** 2026-08-11

**Authors:** Andrew P. Leynes, Jonathan Andreas Saenger, Anna Weber, Thomas Frauenfelder, Andreas Boss

**Affiliations:** 1 Diagnostic and Interventional Radiology, University Hospital Zurich, University Zurich, 8091 Zurich, Switzerland; andrew.leynes@usz.ch (A.P.L.); thomas.frauenfelder@usz.ch (T.F.); andreas.boss@gzo.ch (A.B.); 2 b-rayZ, 8952 Schlieren, Switzerland; 3 Institute of Diagnostic and Interventional Radiology, GZO Regional Health Center, 8620 Wetzikon, Switzerland; anna.weber@gzo.ch

**Keywords:** artificial intelligence, breast cancer, mammography, breast computed tomography

## Abstract

**Purpose**: Breast cancer screening relies primarily on two-dimensional (2D) digital mammography, which is limited by tissue superposition and compression-related patient discomfort. Dedicated spiral breast computed tomography (BCT) overcomes these limitations by providing isotropic, fully three-dimensional (3D) volumetric images without compression. However, translating artificial intelligence (AI) advancements to 3D BCT lesion detection is hindered by the scarcity of large-scale annotated 3D datasets and substantial computational constraints. The purpose of this study is to evaluate the feasibility of repurposing a pre-trained 2D mammography AI model for 3D BCT malignancy detection without dedicated model retraining. **Methods**: In this exploratory proof-of-concept study, the dataset comprised BCT examinations of female patients with malignant lesions. We developed a slab projection framework that systematically rotates the BCT volume and extracts overlapping tissue slabs to simulate mammography-like 2D images compatible with the input requirements of the pre-existing 2D mammography model. The 2D inferences were subsequently back-projected to their original 3D spatial coordinates and aggregated via a consensus voting scheme. Optimization and performance evaluation were performed on the same dataset to establish an internal baseline. Object-level detection performance was assessed using the F1 score, and segmentation quality was evaluated using the Dice coefficient. **Results**: Application of the proposed framework enabled 3D lesion localization in BCT volumes without requiring domain-specific training. An optimal configuration was found with a sparser angular sampling combined with medium slab thickness (45° angular spacing, 50-voxel thickness), which achieved an F1 score of 0.346, with 48.3% precision and 26.9% recall. Evaluation of segmentation quality corroborated these findings, yielding a maximum mean Dice coefficient of 0.336 for the 45°/30-voxel configuration. **Conclusions**: This exploratory proof-of-concept demonstrates the technical feasibility of adapting a pre-trained 2D mammography AI model to volumetric BCT without architectural modifications or retraining. The reported performance represents an internal same-dataset baseline under substantial domain shift and should not be interpreted as generalizable or clinically deployable performance. These findings provide a foundation for future domain-adapted models and independent external validation of AI-based tumour detection in breast CT.

## Simple Summary

Breast CT provides three-dimensional breast imaging without compression but lacks the large annotated datasets required for dedicated AI development. In this proof-of-concept study, we adapted a pre-trained 2D mammography AI model for 3D breast CT lesion detection without additional training. The framework generated multi-angle projections from breast CT volumes, applied the mammography model to each projection, and combined predictions through back-projection and consensus voting. The approach successfully localized malignant lesions, demonstrating the feasibility of transferring existing mammography AI tools to breast CT and providing a foundation for optimization and validation.

## 1. Introduction

Breast cancer (BC) remains a leading cause of cancer-related death among women [1,2]. BC screening programs enable early detection and have been shown to reduce both morbidity and mortality from breast cancer [3,4].

BC screening programs are widely implemented across many countries, with two-view digital mammography (DM) serving as the standard technique due to its low radiation dose and established clinical utility [5]. Beyond the discomfort associated with the required breast compression [6], a principal limitation of DM is its two-dimensional nature: tissue superposition may obscure lesions, reduce sensitivity, or create the appearance of non-existent abnormalities, lowering specificity. Sensitivity is further compromised in dense breast tissue [7], and the presence of breast implants can additionally hinder image acquisition and interpretation [8]. Artificial intelligence (AI), particularly deep learning, has become an important tool for lesion detection in mammography [9,10,11]. Trained on large datasets, these systems can reliably identify masses, calcifications, and architectural distortions. AI can support radiologists by highlighting suspicious regions and reducing perceptual errors, thereby improving diagnostic efficiency. However, careful validation by the radiologist remains essential [12,13].

A recent advancement in breast imaging is the introduction of spiral breast CT (BCT) systems, which provide three-dimensional imaging while eliminating the need for breast compression [14]. Breast CT imaging provides volumetric data with superior depth information compared to conventional mammography [15]. This raises the question of whether advances in artificial intelligence established in mammography can be translated to breast CT imaging. The transition from 2D to 3D lesion detection presents several major challenges, including the limited availability of large-scale annotated 3D breast CT datasets, substantially higher computational demands for training 3D neural networks, and the considerable resources required for developing, retraining, and validating dedicated 3D models [16,17]. Therefore, our framework repurposes an existing mammography-trained lesion detector for breast CT without retraining or access to annotated breast CT training data, unlike previous multi-view aggregation approaches that are typically developed and trained directly on tomosynthesis, CBCT, or other volumetric datasets.

The aim of this study was to evaluate the feasibility of adapting a mammography-trained lesion detection model to breast CT without retraining. Using a multi-angle slab projection framework, we systematically investigated how projection geometry and slab configuration influence detection performance and identified parameter settings that optimize performance in this cross-modality adaptation setting.

## 2. Materials and Methods

### 2.1. Study Population and Design

The local ethics committee approved the prospective study for examination of female patients in a breast CT imager (BASEC-Nr. 2018-01694). Furthermore, the retrospective evaluation of the obtained data using techniques of Artificial Intelligence was approved by the local ethics committee (BASEC-Nr. 2021-01095). All study procedures were conducted in accordance with institutional guidelines, applicable federal regulations, and the Declaration of Helsinki. The study population comprised female patients who had provided informed research consent for study participation and had undergone at least one Breast-CT examination. Examinations of transgender women (male-to-female) were excluded to ensure a homogeneous study population. A total of 25 image volumes from 23 female patients were included in the study. No additional patient-level information, histopathological data, or detailed cohort characteristics were available for analysis.

### 2.2. Breast CT Dataset Acquisition

Breast CT (BCT) image datasets were acquired using a dedicated BCT scanner (nu:view, AB-CT–Advanced Breast-CT GmbH, Erlangen, Germany) without the use of contrast medium in a diagnostic setting. Image acquisition was performed with a fan-beam X-ray source coupled to a CdTe photon-counting detector (PCD; Direct Conversion, Danderyd, Sweden). The scan protocol consisted of one initial circular rotation followed by a transition to a spiral trajectory, with 2000 projections acquired per rotation. The relatively narrow X-ray cone employed in the BCT system reduces scattered radiation, thereby enabling higher image contrast at the same radiation dose compared with conventional cone-beam CT scanners. The BCT-specific PCD, featuring a pixel size of (100 µm)^2^, directly converts incident X-ray photon energy into electrical charge, eliminating intermediate light generation and thus preserving spatial resolution. The X-ray tube voltage was fixed at 60 kVp, and the tube current was set to 32 mA for all examinations.

### 2.3. Ground Truth Generation

The ground-truth generation employed in this study comprises three stages:

Manual Contour Delineation: A board-certified radiologist with more than 25 years of experience in breast imaging manually annotated region-of-interest (ROI) contours at three anatomically significant axial slices for each lesion: the superior extent (start slice), the plane of maximum cross-sectional area (middle slice), and the inferior extent (end slice).

Volumetric Interpolation: Between manually annotated slices, lesion boundaries were interpolated using linear interpolation to generate a preliminary 3D segmentation mask.

Deep Learning-Based Refinement: The interpolated masks were subsequently refined using MedSAM2′s 3D propagation mode [18]. This approach utilized the manually annotated slices as initialization prompts and propagated the segmentation bidirectionally through the volume, leveraging 3D contextual information to refine boundary delineation. The final output consisted of validated ground truth volumes with per-voxel labels (0 = background, 1 = benign, 2 = malignant).

### 2.4. 3D Breast CT Adaptation to 2D Mammography Model: Slab Projection Method and AI Inference

**Figure 1:**
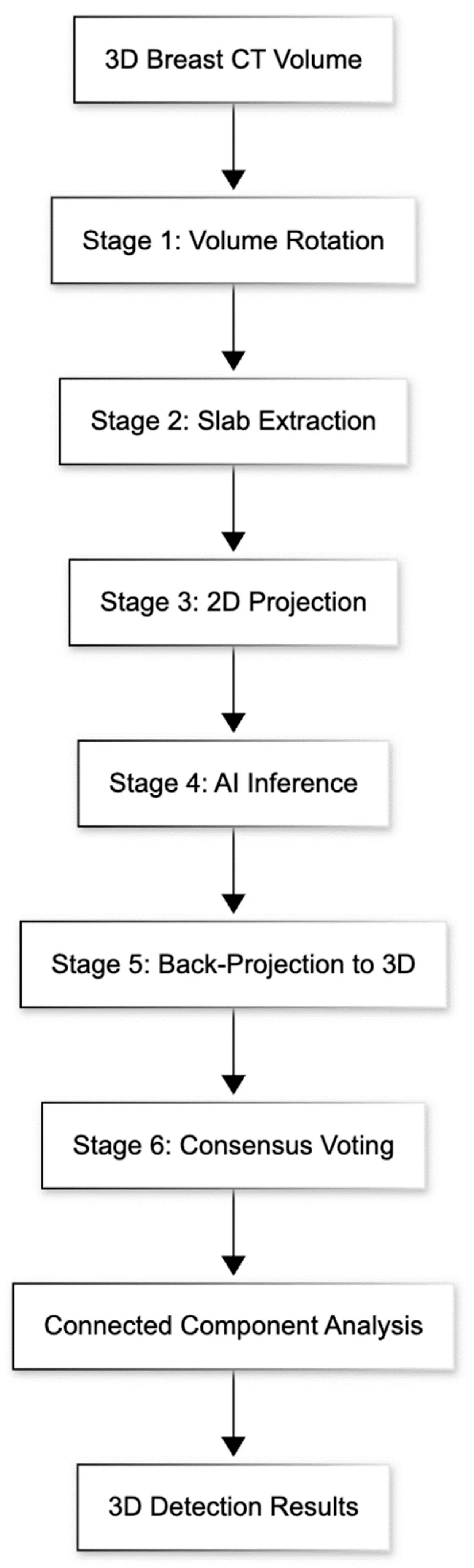
Forward Projection Pipeline.

**Figure 2:**
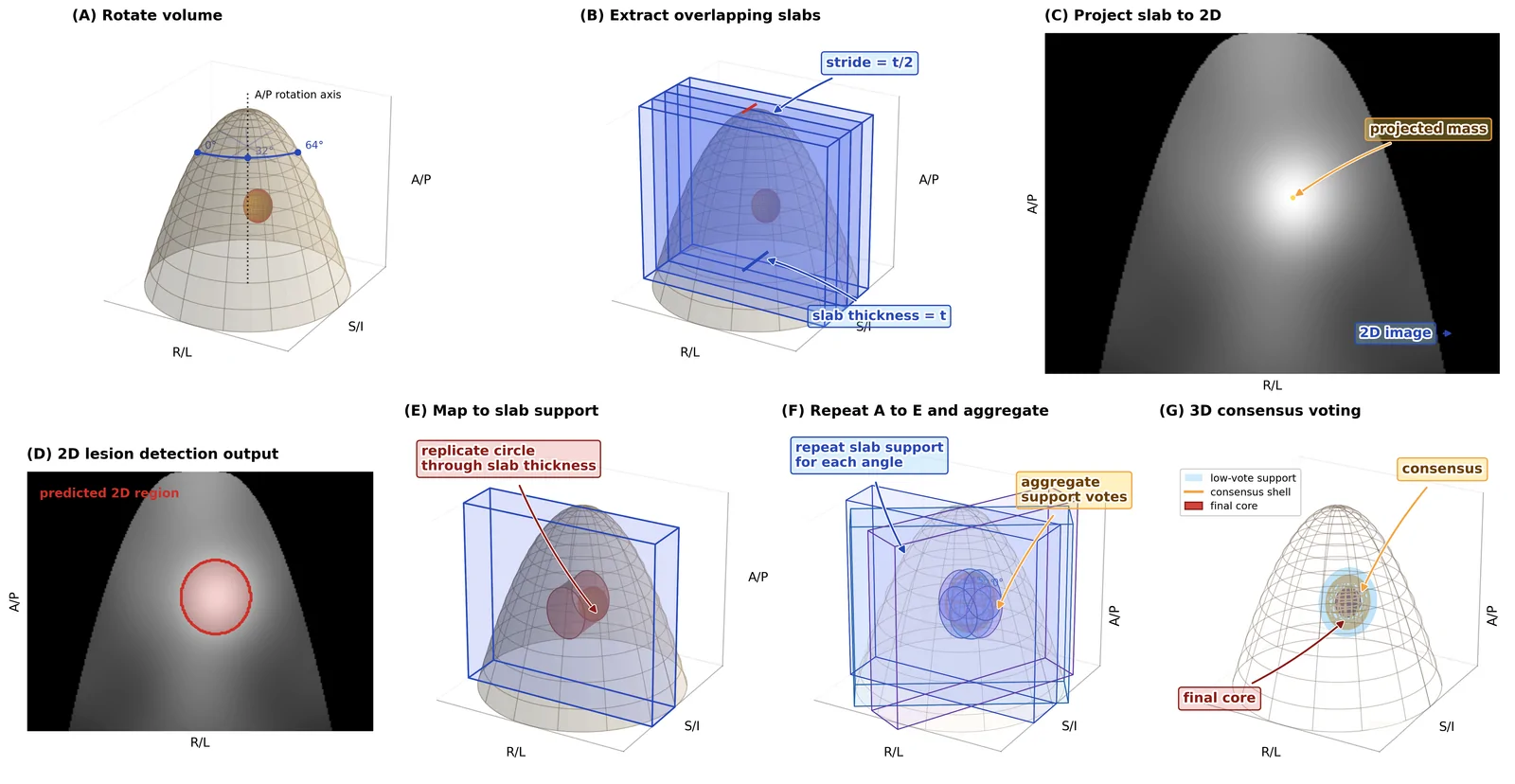
Schematic representation of the annotated multi-angle slab projection workflow: (**A**) rotation of the breast CT volume; (**B**) extraction of overlapping slabs; (**C**) 2D projection of each slab; (**D**) lesion detection output on the slab projection; (**E**) mapping of 2D detections back to their originating slab support in 3D; (**F**) repetition across rotation angles with aggregation of slab-level detections; and (**G**) final 3D consensus voting to obtain lesion candidates.

The core contribution of this work is the limited-thickness slab projection method, which resamples the 3D volume to generate 2D projection images compatible with the pre-trained 2D mammography lesion detection model. The complete processing pipeline is illustrated in Figure 1 and Figure 2 summarizes the workflow from volume rotation and overlapping slab generation through 2D projection, AI detection, back-projection to slab support, repetition across viewing angles, and final 3D consensus voting.

#### 2.4.1. Stage 1: Volume Rotation

The 3D breast CT volume was rotated around the anterior–posterior (A/P) axis to sample multiple viewing directions while preserving the native voxel grid for subsequent inverse mapping. Rotation angles spanned 0° to 180°, and the angular spacing (10°, 15°, 30°, or 45°) determined the number of viewpoints evaluated for each volume. Because opposite directions are redundant for the projection geometry used here, a hemispherical sampling scheme was sufficient.

#### 2.4.2. Stage 2: Slab Extraction

For each rotated volume, thick slabs are extracted along the superior-inferior (S/I) axis at different positions. Slabs are extracted with 50% overlap (stride = thickness/2) to ensure lesions near slab boundaries are adequately captured in adjacent slabs. Breast CT images had an isotropic voxel size of 0.3 mm. Slab thickness was defined in voxels throughout the analysis, with evaluated thicknesses of 5, 10, 20, 30, 50, and 100 voxels corresponding to physical slab thicknesses of 1.5, 3, 6, 9, 15, and 30 mm, respectively. The configurable slab thickness (5, 10, 20, 30, 50, or 100 voxels) governs the trade-off between depth resolution and signal-to-noise characteristics.

#### 2.4.3. Stage 3: Two-Dimensional Projection

Each 3D slab is collapsed to a 2D image through mean intensity projection along the slab thickness (rotated to be the S/I axis):

$$projection[ap,\;rl]=\frac{1}{N}\sum_{i=si\_start}^{si\_end}slab[ap,\;i,\;rl]$$

where N denotes the slab thickness. This simulates an X-ray projection along a section of tissue.

#### 2.4.4. Stage 4: AI Inference

The pre-trained 2D breast lesion detection model (b-rayZ b-box) processes each projection independently. For each detected lesion, the model outputs: (1) normalized 2D bounding box coordinates, (2) contour points defining the lesion boundary, (3) BI-RADS classification (categories 2–5), and (4) detection confidence score.

#### 2.4.5. Stage 5: Back-Projection to 3D Coordinates

Each 2D detection is transformed back to the original 3D volume coordinate system through: (1) rasterization of the 2D contour to a binary mask, (2) replication across the S/I dimension within original slab bounds, and (3) application of inverse rotation to recover original coordinates to map the 2D detection back into the correct 3D spatial location with nearest neighbor resampling.

#### 2.4.6. Stage 6: Repetition Across Angles and Aggregation

Stages 1–5 were repeated for every rotation angle included in the selected angular spacing. This produced a set of overlapping slab-level detections from multiple viewing directions for each breast CT volume.

#### 2.4.7. Stage 7: Consensus Voting and Detection Merging

Multiple 2D detections are aggregated through consensus voting: vote accumulation per voxel, confidence aggregation, class-specific vote tracking, consensus thresholding, and connected component analysis to yield final detections.

Each transformed detection contributes a binary 3D segmentation mask, a predicted BIRADS class, and a confidence score. For every voxel in the original volume, the pipeline accumulates:

- A total “vote_counts” volume, counting how many detections include that voxel;
- A “confidence_sums” volume, adding each contributing detection’s confidence score at that voxel;
- Separate class-specific vote volumes, tracking how many votes each BIRADS class receives at each voxel.

A final merged segmentation mask is created by thresholding the vote count volume: a voxel is retained only if it receives at least 3 votes.

The thresholded mask is then split into individual candidate findings using 3D connected component analysis. For each connected component, the pipeline computes a 3D bounding box in [A/P, S/I, R/L] voxel coordinates, assigns a final BIRADS class by majority vote across the class-specific vote volumes within that component, and records summary statistics including average consensus votes, total contributing detections, region size in voxels, and average confidence. Average confidence is calculated as the summed confidence over the component divided by the summed vote count, such that it reflects the mean confidence of detections that contributed to the retained voxels.

The final outputs include the merged 3D segmentation mask, voxel-wise vote/confidence volumes, and a list of final detections containing each component’s bounding box, majority-vote BIRADS class, consensus vote statistics, detection count, region size, and average confidence. The final BIRADS classes are compressed to benign/malignant binary classification as follows: BIRADS 2/3 → benign, BIRADS 4/5 → malignant.

### 2.5. Systematic Optimization of Slab Projection Parameters

To characterize the performance landscape and identify optimal operating parameters for the slab projection adaptation, a systematic grid search was conducted across several combinations of angular spacing and slab thickness.

Angular Spacing Configurations:

1. 10°: 18 viewing angles (0°, 10°, 20°, …, 170°)
2. 15°: 12 viewing angles (0°, 15°, 30°, …, 165°)
3. 30°: 6 viewing angles (0°, 30°, 60°, …, 150°)
4. 45°: 4 viewing angles (0°, 45°, 90°, 135°)

Slab Thickness Configurations:

1. 5 voxels thick
2. 10 voxels thick
3. 20 voxels thick
4. 30 voxels thick
5. 50 voxels thick
6. 100 voxels thick

This yields 24 distinct parameter configurations. For each configuration, slab projection and lesion detection inference were performed on all available breast CT volumes, and predictions were compared against ground truth segmentations. As a complementary reference analysis, we additionally defined a projection-level 2D slab evaluation in which detections are assessed on the generated slab projections before 3D back-projection and consensus merging. This auxiliary analysis was intended to isolate the contribution of the 3D aggregation stage relative to the underlying 2D detector, without changing the detector itself, and any corresponding results are reported separately where included.

### 2.6. Statistics

Predicted and ground truth lesions were matched using 3D Intersection-over-Union (IoU) with a threshold of 0.15. A greedy matching algorithm sorted candidate pairs by descending IoU and ascending centroid distance, assigning highest-scoring pairs while preventing duplicate matches. For assessment of object-level detection metrics, precision, recall, and F1 score were computed. Results were evaluated across confidence thresholds from 0.0 to 0.95. Segmentation quality was assessed with the Dice coefficient between predicted segmentation masks and ground truth. For assessment of image-level metrics, the worst-case prediction was taken as the prediction for the whole image: if both benign and malignant objects were predicted, the image will have a malignant prediction. Lesion-level recall was calculated as the proportion of reference lesions that were successfully detected by the proposed framework. Maximum recall was defined as the highest achievable recall on the precision-recall curve, corresponding to the minimum confidence threshold. The image-level prediction is then binarized to normal/benign vs malignant.

For the primary 3D object-level metrics, 95% confidence intervals for precision, recall, and F1 score were computed by patient-clustered non-parametric bootstrap resampling. For the auxiliary 2D projection-level evaluation, metrics were treated as class-agnostic 2D object-detection metrics on the generated slab projection images, before 3D back-projection or consensus voting. For each angle/thickness combination, 95% confidence intervals for 2D object precision, object sensitivity/recall, object F1, and AP were computed using nonparametric bootstrap resampling of slab projection images (5000 replicates) at that combination’s best-F1 confidence threshold and IoU = 0.3 for threshold-specific metrics. For image-level 3D sensitivity, specificity, precision, and accuracy, 95% confidence intervals were computed using the Wilson method. Image-level AUC 95% confidence intervals were estimated by bootstrap resampling of cases/series (5000 replicates) and recomputing the threshold-sweep ROC AUC from sensitivity and specificity across confidence thresholds for each configuration. For mean Dice coefficients, 95% confidence intervals were computed from the t distribution across volumes.

## 3. Results

### 3.1. Optimization Precision-Recall Trade-Offs, Best Performing Configurations and Effect of Slab Thickness

**Figure 3:**
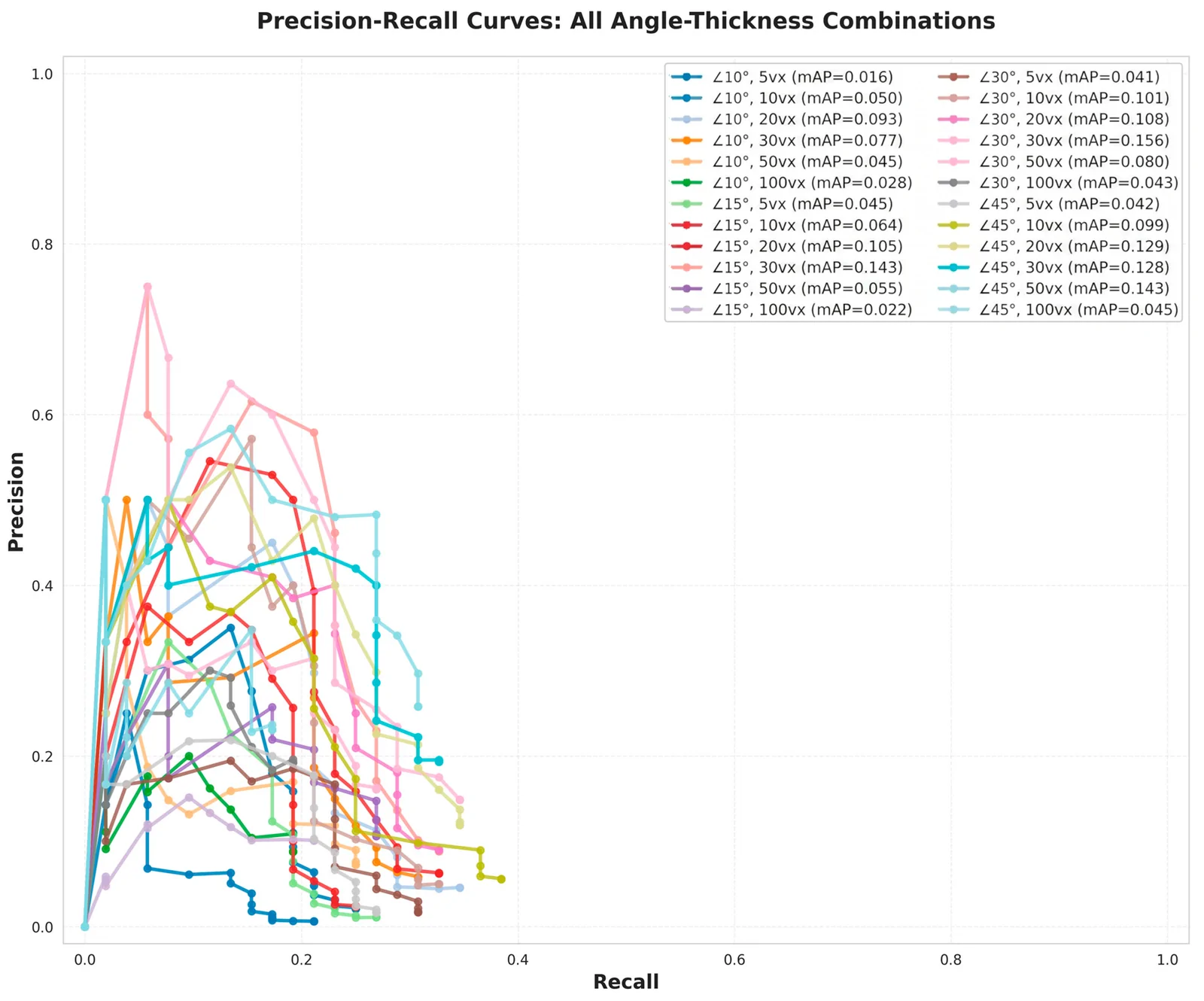
Precision-recall curves across all parameter configurations and the corresponding mean-average precision metric.

**Figure 4:**
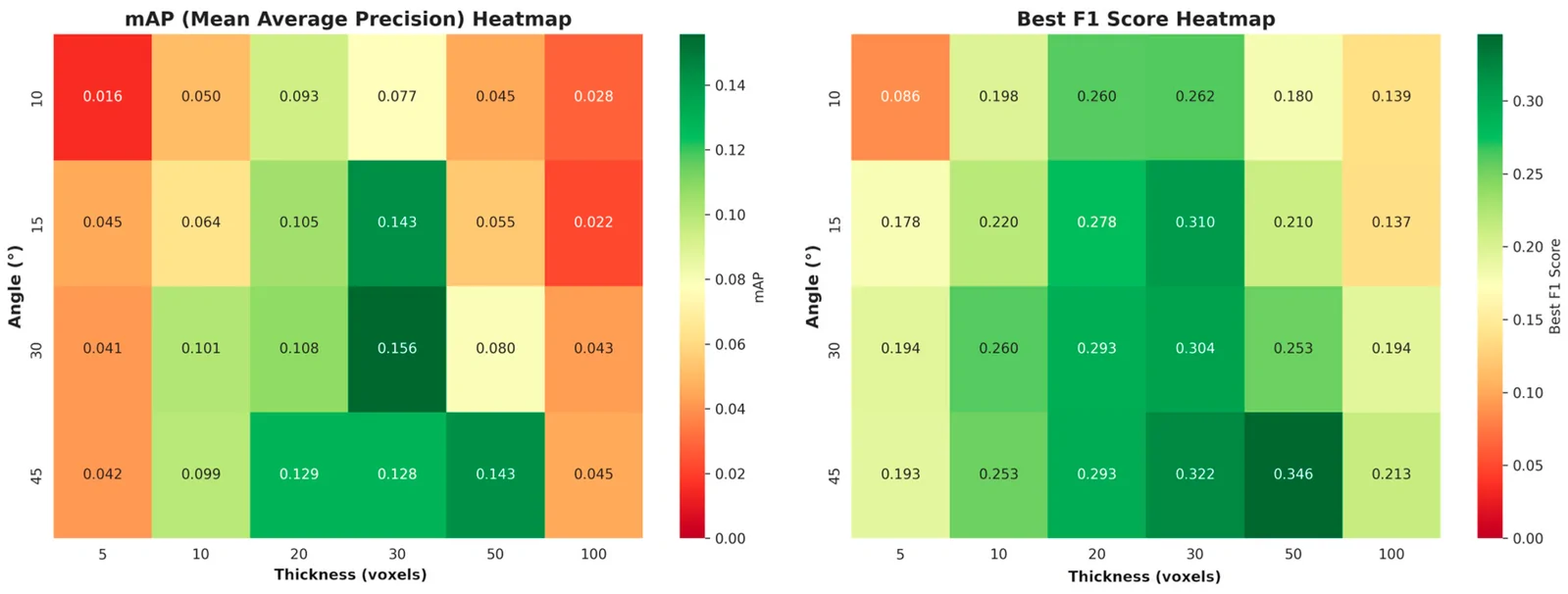
Performance heatmaps across the parameter space.

Analysis of the 24 parameter configurations revealed systematic relationships between parameter choices and detection performance. Table 1 presents performance metrics for all parameter configurations at their optimal confidence thresholds. Precision-recall curves for all evaluated parameter configurations are shown in Figure 3. The optimal configuration (45° angular spacing, 50-voxel slab thickness) achieved an F1 score of 0.346 at a confidence threshold of 0.35, with precision of 48.3% (95% CI, 35.7–64.7%), recall of 26.9% (95% CI, 14.3–51.7%), and F1 of 34.6% (95% CI, 21.1–54.0%), in Figure 4 a performance heatmap across the parameter space is depicted. Sparser angular sampling (45°) outperformed denser sampling (10°) in terms of F1 score, as shown in Table 2. This finding suggests that denser angular sampling may introduce redundant or conflicting detections that reduce precision without proportional recall gains. Medium slab thicknesses (30–50 voxels) consistently outperformed both thin (5–10 voxels) and very thick (100 voxels) configurations:

• Thin slabs (5–10 vx): Best F1 range 0.086–0.253; high false positive rates likely due to noise;
• Medium slabs (20–50 vx): Best F1 range 0.180–0.346; better signal-to-noise balance;
• Thick slabs (100 vx): Best F1 range 0.136–0.213; reduced sensitivity due to structure masking.

**Table 1:** Top 10 3D detection configurations ranked by F1 score at their optimal confidence thresholds based on best F1 score.

Angle (°)	Thickness (vx)	Threshold	Precision (95% CI)	Recall (95% CI)	F1 (95% CI)	Max Recall
45	50	0.35	48.3% (35.7–64.7%)	26.9% (14.3–51.7%)	34.6% (21.1–54.0%)	0.308
45	30	0.40	40.0% (26.2–59.4%)	26.9% (14.3–51.7%)	32.2% (19.5–51.7%)	0.327
15	30	0.50	57.9% (38.9–78.6%)	21.2% (10.2–42.9%)	31.0% (17.0–51.1%)	0.327
30	30	0.45	44.4% (31.0–59.1%)	23.1% (11.7–45.7%)	30.4% (17.6–48.4%)	0.346
45	20	0.55	47.8% (31.4–70.6%)	21.2% (10.5–42.4%)	29.3% (16.7–47.3%)	0.346
30	20	0.50	40.0% (24.2–60.0%)	23.1% (11.7–45.7%)	29.3% (16.7–47.6%)	0.327
15	20	0.55	50.0% (32.0–75.0%)	19.2% (9.1–39.5%)	27.8% (14.5–48.0%)	0.327
10	30	0.50	34.4% (20.9–55.0%)	21.2% (10.2–42.9%)	26.2% (15.2–40.6%)	0.308
10	20	0.55	40.0% (25.0–57.1%)	19.2% (9.1–39.5%)	26.0% (14.1–42.1%)	0.346
30	50	0.35	31.4% (17.6–50.0%)	21.2% (10.6–41.9%)	25.3% (14.1–41.8%)	0.269

**Table 2:** Best F1 scores for the best configurations for different angle spacings.

Angle (°)	Best F1 (95% CI)	Best Config
45	34.6% (21.1–54.0%)	50 vx
30	30.4% (17.6–48.4%)	30 vx
15	31.0% (17.0–51.1%)	30 vx
10	26.2% (15.2–40.6%)	30 vx

The complete set of 3D detection metrics with 95% confidence intervals is provided in Supplementary Table S1.

### 3.2. Segmentation Quality

**Figure 5:**
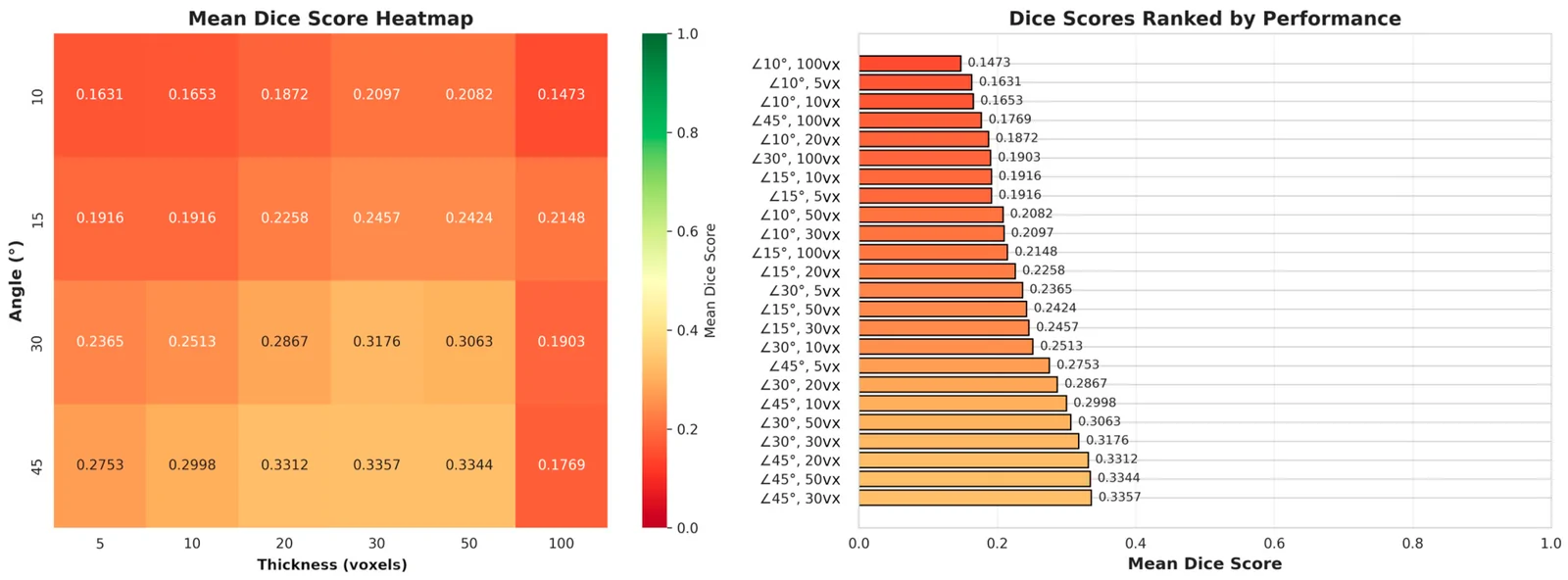
Dice coefficient analysis.

Dice coefficient analysis (Figure 5) corroborates the detection findings, with sparser angular sampling achieving higher spatial overlap with ground truth. The complete configurations are shown in Supplementary Table S2. The 45°/30-voxel configuration achieved the highest mean Dice coefficient (0.336; 95% CI, 0.217–0.455), closely followed by 45°/50-voxel (0.334; 95% CI, 0.205–0.464).

### 3.3. Image Level Classification

**Figure 6:**
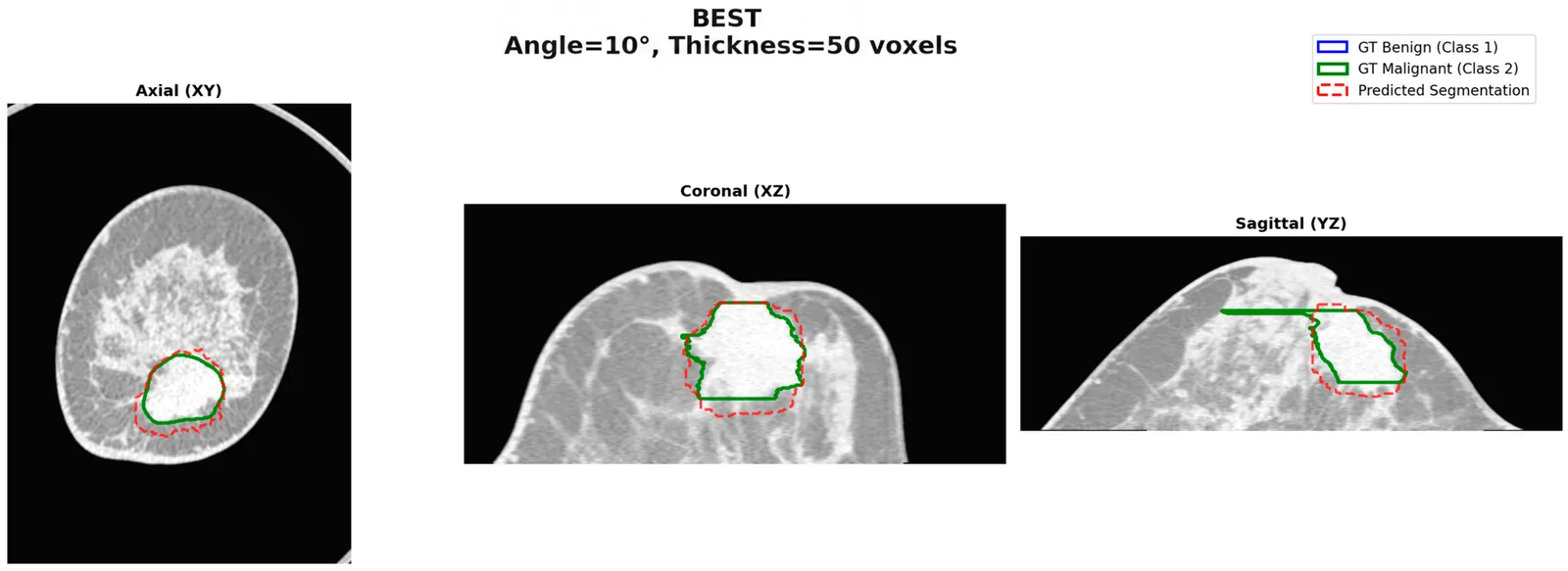
Example for the detection of breast cancer (angle = 10°, thickness = 50 vx).

Supplementary Figure S1 presents ROC curves for volume-level malignancy classification and a matrix for image-level classification for different configurations is displayed in Supplementary Figure S2. Image-level performance achieves a maximum AUC of 0.720 (95% CI, 0.000–0.841) for distinguishing between malignant versus non-malignant/benign cases with the optimal parameter configuration of 5 vx thickness and an angle of 30°. No particular pattern regarding optimization was found, e.g., the second highest AUC was achieved for 30 vx thickness and an angle of 45°. A typical example of a well-detected case is provided in Figure 6. Complete image-level classification metrics, AUC values, and 95% confidence intervals are provided in Supplementary Tables S3 and S4.

### 3.4. 2D Projection-Image-Level Evaluation

As an auxiliary analysis before 3D back-projection and consensus merging, we evaluated the generated 2D slab projections directly as a class-agnostic object-detection task across all 24 angle/thickness combinations; the full summary is provided in Supplementary Table S5. The best 2D object-detection performance was obtained for 45° angular spacing and 10-voxel slab thickness at a confidence threshold of 0.25, with object precision of 36.0% (95% CI, 34.2–38.0%), object sensitivity/recall of 15.8% (95% CI, 14.6–17.1%), and F1 of 22.0% (95% CI, 20.6–23.5%). This same configuration also achieved the highest AP@IoU 0.3 (10.5%; 95% CI, 9.5–11.7%) and AP@IoU 0.5 (4.8%; 95% CI, 4.2–5.6%).

## 4. Discussion

We investigated the feasibility of adapting pre-trained 2D mammography lesion detection models for 3D breast CT using a multi-angle slab projection and consensus voting approach. We demonstrated that this framework enables transfer of detection capabilities to volumetric data without model retraining and identified optimal parameter configurations that balance detection performance and computational efficiency.

The principal findings are:

• Detection performance: The optimal configuration (45° angular spacing, 50-voxel slab thickness) achieved an F1 score of 0.346, with 48.3% precision and 26.9% recall at the optimal threshold.
• Parameter relationships: Contrary to initial hypotheses, sparser angular sampling (45°) outperformed denser configurations (10°), likely due to reduced accumulation of conflicting or noisy detections. Medium slab thicknesses (30–50 voxels) provided the best balance between signal quality and depth resolution.
• Segmentation quality: The optimal configurations achieved mean Dice coefficients of 0.33–0.34, indicating moderate spatial agreement with ground truth segmentations.

In general, AI-assisted interpretation has been shown to reduce recall rates and radiologist workload while maintaining diagnostic performance [19]. Previous studies have demonstrated that AI systems combining multiple imaging modalities, such as 2D mammography and 3D tomosynthesis, can improve diagnostic performance by leveraging complementary information across [20,21]. These findings highlight the clinical relevance of developing efficient and transferable AI solutions for emerging imaging modalities such as breast CT. However, such approaches typically depend on large-scale annotated datasets and dedicated training pipelines to achieve robust generalizability, which are currently not available for breast CT. Mendel et al. previously demonstrated that pre-trained 2D mammography CNNs can be repurposed for digital breast tomosynthesis by extracting deep features from DBT key slices and combining standard views using soft-voting classifiers, resulting in improved lesion classification compared with conventional full-field digital mammography. To our knowledge, comparable evidence for breast CT is currently lacking [22]. While multi-view aggregation strategies have been explored in DBT and other volumetric imaging modalities, the primary contribution of the present study is the zero-shot adaptation of a mammography-trained lesion detection model to breast CT without retraining or breast CT-specific annotations.

The moderate AI performance observed in this study likely reflects the substantial domain shift between the original training domain, mammography, and the target domain, breast CT. Differences in acquisition geometry, particularly the absence of breast compression in CT and the use of volumetric rather than projection imaging, lead to modality-dependent variations in tissue contrast and lesion appearance [23]. While the achieved AUC suggests that zero-shot transfer learning is feasible, the modest overall performance and low lesion-level recall currently limit its clinical utility. Therefore, the present approach should be considered an exploratory proof-of-concept rather than a tool ready for integration into routine radiological workflows.

The similar attenuation of glandular tissue and lesions in breast CT may further impair lesion detection, particularly in dense breasts [24,25]. While extensive literature exists comparing AI performance in 2D mammography and 3D tomosynthesis, demonstrating improved lesion detection with volumetric imaging, direct comparisons between mammography and breast CT in the context of AI-based detection remain limited. Digital breast tomosynthesis (DBT), a quasi-3D imaging technique, has been shown to improve lesion detection compared to conventional 2D mammography, with higher sensitivity, improved specificity, and increased cancer detection rates, particularly in dense breast tissue [20]. The concurrent use of an accurate DBT AI system has been shown to further improve cancer detection efficacy, including increases in AUC, sensitivity, and specificity, along with reductions in recall rate and reading time [11,26]. The proposed method in this manuscript enables the transfer of pre-trained 2D mammography models to 3D breast CT using a projection-based aggregation strategy that combines predictions across multiple views, thereby reducing uncertainty and stabilizing detection performance [11].

Breast CT provides true three-dimensional imaging without tissue superposition or compression, improving lesion detection, particularly in dense breast tissue where overlapping structures in conventional mammography can reduce diagnostic sensitivity [27,28]. Combining predictions across multiple views or modalities enhances detection performance by leveraging complementary information and reducing uncertainty [19]. By offering volumetric data with consistent depth information, BCT may also further enhance lesion characterization [16]. However, these advantages also introduce new challenges for image analysis, as lesion detection must be performed in a fully three-dimensional context rather than on projection images. Feature extraction is performed across high-dimensional volumetric data with large feature spaces, requiring dimensionality reduction and complex processing pipelines [22]. Leveraging full 3D breast CT data substantially increases computational demands and interpretation time [19,29,30]. In our framework, inference time scaled directly with the number of slab projections, with exhaustive configurations requiring several hours for complete dataset analysis. Notably, reducing angular sampling and increasing slab thickness substantially decreased computational burden, while also improving detection performance. The optimal configuration (45° angular spacing, 50-voxel slab thickness) achieved this balance, suggesting that efficient sampling strategies may mitigate the trade-off between computational cost and diagnostic performance. Further optimization of the processing pipeline, including batched inference and GPU acceleration, may enhance scalability and facilitate clinical implementation. The consensus voting mechanism offers several key advantages. By requiring consistency across multiple views, it reduces isolated false-positive detections that may arise in single projections. At the same time, it enables aggregation of detection confidence across different viewpoints, leading to more robust and reliable predictions. Furthermore, the approach allows flexible adjustment of the operating point through threshold selection, making it adaptable to different clinical requirements and performance trade-offs.

Several limitations should be acknowledged. The relatively small sample size of only 25 cases limits generalizability, and the absence of benign lesions restricts evaluation of specificity and performance in benign cases. Additionally, ground truth annotations were provided by a single reader, precluding assessment of inter-observer variability. Finally, the absence of domain-specific training likely constrains the achievable performance ceiling. Despite these challenges, the proposed approach establishes a functional baseline for 2D-to-3D adaptation without requiring any model retraining.

In addition, the evaluation was limited by the absence of an external validation cohort and by the use of parameter optimization and performance assessment within the same dataset. Therefore, the reported performance should be interpreted as baseline proof-of-concept performance rather than as an estimate of generalizable clinical performance.

Future work should aim to reduce domain shift through targeted fine-tuning of the 2D model on slab projections, potentially leading to substantial improvements in detection performance. Larger, more diverse datasets are required to validate generalizability, and multi-reader studies could provide more robust reference standards. In parallel, computational optimization will be essential to enable practical deployment in clinical workflows.

## 5. Conclusions

In conclusion, this work presents an exploratory, non-clinically deployable proof-of-concept framework for adapting 2D mammography AI models to 3D breast CT lesion detection through systematic multi-angle slab projections and consensus-based aggregation. While the approach successfully transfers 2D detection capability to the 3D domain without retraining, the observed performance (F1 = 0.346) should be interpreted as an internal baseline obtained under substantial domain shift between mammography and breast CT rather than as an estimate of clinically achievable performance. The identified optimal parameters (45° angular spacing, 50 voxel slab thickness) establish a reference framework for future domain-adapted and fine-tuned models aimed at overcoming this performance gap. Overall, this study demonstrates the technical feasibility of repurposing existing 2D medical imaging AI models for volumetric breast CT and provides a benchmark for future method development and external validation.

Key Points

• Proof-of-Concept for 2D to 3D Domain Adaptation: This investigation demonstrates the feasibility of “zero-shot” domain adaptation, showing that 2D mammography AI models can localize malignancies in fully 3D breast CT with an adaptation approach. This approach circumvents the critical bottleneck of limited annotated 3D datasets and the high computational costs of 3D model development from scratch.
• Optimization of Volumetric Sampling: Systematic analysis revealed that sparser angular sampling (45°) and medium-thickness slab projections (30–50 voxels) yield optimal 3D detection. Contrary to initial hypotheses that denser angular sampling would yield better performance, this configuration best balances signal quality with depth resolution while actively mitigating the accumulation of noisy or conflicting 2D detections within the 3D space.
• Quantifying the Modality Gap: While the framework effectively transfers 2D detection capabilities without retraining, the moderate baseline diagnostic performance (F1 = 0.346) highlights and quantifies a substantial domain shift between compressed projection mammography and uncompressed volumetric CT.
• Foundation for Accelerated AI Development: By establishing a functional algorithmic baseline, this methodology validates the repurposing of existing 2D algorithms for emerging 3D. It provides a defined quantitative starting point for future targeted fine-tuning, offering a highly resource-efficient pathway to clinical integration.

## Data Availability

The datasets presented in this article are available upon reasonable request.

## Supplementary Materials

The following supporting information can be downloaded at https://www.mdpi.com/article/10.3390/cancers18162585/s1, Supplementary Table S1: Complete 3D object-detection; Supplementary Table S2: Complete 3D Dice; Supplementary Table S3: 3D image-level classification at best F1; Supplementary Table S4: 3D image-level Benign vs. Malignant Classification AUC; Supplementary Table S5: 2D slab-projection object-detection performance; Supplementary Figure S1: ROC curves for volume-level malignancy classification; Supplementary Figure S2: Matrix for image-level classification for different configurations.
