# Supplementary material for: Zero-Shot Domain Adaptation of 2D Mammography Lesion Detection Model for 3D Breast CT Malignancy Detection
Source: Cancers (Basel). 2026 Aug 11;18(16):2585. doi: 10.3390/cancers18162585 (PMC13511489; doi:10.3390/cancers18162585)
Supplement: Supplementary file 1 [file cancers-18-02585-s001.zip › Supplementary Table S1_ Complete 3D object-detection supplementary table.pdf]

Supplementary Table S1: Complete 3D object-detection

One row per angle/thickness configuration at the best-F1 detection threshold.

angle_deg	thickness_vx	best_threshold	tp	fp	fn	precision_with_ci	recall_with_ci	f1_with_ci
10	5	0.6	7	104	45	6.3% (95% CI 2.5%-11.9%)	13.5% (95% CI 5.7%-29.0%)	8.6% (95% CI 3.6%-15.6%)
10	10	0.65	8	21	44	27.6% (95% CI 13.8%-50.0%)	15.4% (95% CI 6.6%-33.3%)	19.8% (95% CI 9.1%-38.1%)
10	20	0.55	10	15	42	40.0% (95% CI 25.0%-57.1%)	19.2% (95% CI 9.1%-39.5%)	26.0% (95% CI 14.1%-42.1%)
10	30	0.5	11	21	41	34.4% (95% CI 20.9%-55.0%)	21.2% (95% CI 10.2%-42.9%)	26.2% (95% CI 15.2%-40.6%)
10	50	0.35	10	49	42	16.9% (95% CI 8.3%-33.3%)	19.2% (95% CI 9.5%-38.7%)	18.0% (95% CI 9.1%-34.8%)
10	100	0.15	10	82	42	10.9% (95% CI 5.6%-17.6%)	19.2% (95% CI 9.5%-38.7%)	13.9% (95% CI 7.5%-22.2%)
15	5	0.6	9	40	43	18.4% (95% CI 9.4%-28.3%)	17.3% (95% CI 8.0%-35.7%)	17.8% (95% CI 8.9%-30.6%)
15	10	0.55	10	29	42	25.6% (95% CI	19.2% (95% CI	22.0% (95% CI

angle_deg	thickness_vx	best_threshold	tp	fp	fn	precision_with_ci	recall_with_ci	f1_with_ci
						15.2%-41.9%)	9.1%-39.5%)	11.5%-38.8%)
15	20	0.55	10	10	42	50.0% (95% CI 32.0%-75.0%)	19.2% (95% CI 9.1%-39.5%)	27.8% (95% CI 14.5%-48.0%)
15	30	0.5	11	8	41	57.9% (95% CI 38.9%-78.6%)	21.2% (95% CI 10.2%-42.9%)	31.0% (95% CI 17.0%-51.1%)
15	50	0.35	11	42	41	20.8% (95% CI 11.5%-33.3%)	21.2% (95% CI 10.6%-41.9%)	21.0% (95% CI 11.4%-35.7%)
15	100	0	11	98	41	10.1% (95% CI 4.5%-18.4%)	21.2% (95% CI 8.1%-46.7%)	13.7% (95% CI 6.0%-25.4%)
30	5	0.55	12	60	40	16.7% (95% CI 10.7%-24.2%)	23.1% (95% CI 11.6%-45.2%)	19.4% (95% CI 11.5%-30.0%)
30	10	0.55	10	15	42	40.0% (95% CI 23.8%-61.1%)	19.2% (95% CI 9.1%-39.5%)	26.0% (95% CI 13.5%-44.9%)
30	20	0.5	12	18	40	40.0% (95% CI 24.2%-60.0%)	23.1% (95% CI 11.7%-45.7%)	29.3% (95% CI 16.7%-47.6%)
30	30	0.45	12	15	40	44.4% (95% CI 31.0%-59.1%)	23.1% (95% CI 11.7%-45.7%)	30.4% (95% CI 17.6%-48.4%)
30	50	0.35	11	24	41	31.4% (95% CI	21.2% (95% CI	25.3% (95% CI

angle_deg	thickness_vx	best_threshold	tp	fp	fn	precision_with_ci	recall_with_ci	f1_with_ci
						17.6%-50.0%)	10.6%-41.9%)	14.1%-41.8%)
30	100	0.1	10	41	42	19.6% (95% CI 9.8%-34.0%)	19.2% (95% CI 7.8%-40.6%)	19.4% (95% CI 9.2%-34.5%)
45	5	0.6	11	51	41	17.7% (95% CI 10.0%-27.3%)	21.2% (95% CI 10.6%-41.7%)	19.3% (95% CI 11.2%-31.0%)
45	10	0.55	11	24	41	31.4% (95% CI 19.4%-48.0%)	21.2% (95% CI 10.5%-42.4%)	25.3% (95% CI 14.2%-42.6%)
45	20	0.55	11	12	41	47.8% (95% CI 31.4%-70.6%)	21.2% (95% CI 10.5%-42.4%)	29.3% (95% CI 16.7%-47.3%)
45	30	0.4	14	21	38	40.0% (95% CI 26.2%-59.4%)	26.9% (95% CI 14.3%-51.7%)	32.2% (95% CI 19.5%-51.7%)
45	50	0.35	14	15	38	48.3% (95% CI 35.7%-64.7%)	26.9% (95% CI 14.3%-51.7%)	34.6% (95% CI 21.1%-54.0%)
45	100	0.25	8	15	44	34.8% (95% CI 13.3%-83.3%)	15.4% (95% CI 5.6%-34.4%)	21.3% (95% CI 8.1%-43.1%)
