# Supplementary material for: Zero-Shot Domain Adaptation of 2D Mammography Lesion Detection Model for 3D Breast CT Malignancy Detection
Source: Cancers (Basel). 2026 Aug 11;18(16):2585. doi: 10.3390/cancers18162585 (PMC13511489; doi:10.3390/cancers18162585)
Supplement: Supplementary file 1 [file cancers-18-02585-s001.zip › Supplementary Table S2_ Complete 3D Dice.pdf]

Supplementary Table S2: Complete 3D Dice

angle_deg	thickness_vx	mean_dice_with_ci	std_dice	n	min_dice	max_dice
10	5	0.163 (95% CI 0.086-0.240)	0.185594	25	0	0.691381
10	10	0.165 (95% CI 0.091-0.240)	0.180464	25	0	0.702686
10	20	0.187 (95% CI 0.109-0.265)	0.189187	25	0	0.667876
10	30	0.210 (95% CI 0.125-0.295)	0.205946	25	0	0.699862
10	50	0.208 (95% CI 0.124-0.293)	0.20445	25	0	0.697695
10	100	0.147 (95% CI 0.073-0.222)	0.180139	25	0	0.652376
15	5	0.192 (95% CI 0.104-0.279)	0.21137	25	0	0.735155
15	10	0.192 (95% CI 0.109-0.274)	0.200775	25	0	0.737239

angle_deg	thickness_vx	mean_dice_with_ci	std_dice	n	min_dice	max_dice
15	20	0.226 (95% CI 0.136-0.316)	0.218511	25	0	0.748444
15	30	0.246 (95% CI 0.150-0.342)	0.232893	25	0	0.759149
15	50	0.242 (95% CI 0.144-0.341)	0.237831	25	0	0.734129
15	100	0.215 (95% CI 0.120-0.310)	0.230206	25	0	0.671973
30	5	0.236 (95% CI 0.136-0.337)	0.242786	25	0	0.790105
30	10	0.251 (95% CI 0.152-0.351)	0.241713	25	0	0.795453
30	20	0.287 (95% CI 0.179-0.394)	0.260097	25	0	0.804942
30	30	0.318 (95% CI 0.203-0.432)	0.277592	25	0	0.79342
30	50	0.306 (95% CI 0.184-0.428)	0.29557	25	0	0.775463

angle_deg	thickness_vx	mean_dice_with_ci	std_dice	n	min_dice	max_dice
30	100	0.190 (95% CI 0.084-0.296)	0.256615	25	0	0.764631
45	5	0.275 (95% CI 0.165-0.385)	0.266326	25	0	0.815333
45	10	0.300 (95% CI 0.187-0.412)	0.272633	25	0	0.816855
45	20	0.331 (95% CI 0.214-0.448)	0.283173	25	0	0.787981
45	30	0.336 (95% CI 0.217-0.455)	0.288023	25	0	0.769952
45	50	0.334 (95% CI 0.205-0.464)	0.313124	25	0	0.787948
45	100	0.177 (95% CI 0.067-0.287)	0.26643	25	0	0.815176
