# Supplementary material for: Zero-Shot Domain Adaptation of 2D Mammography Lesion Detection Model for 3D Breast CT Malignancy Detection
Source: Cancers (Basel). 2026 Aug 11;18(16):2585. doi: 10.3390/cancers18162585 (PMC13511489; doi:10.3390/cancers18162585)
Supplement: Supplementary file 1 [file cancers-18-02585-s001.zip › Supplementary Table S3_ Complete 3D image-level classification at best F1.pdf]

Supplementary Table S3: 3D image-level classification at best F1

angle_deg	thickness_vx	selected_threshold	tp	tn	fp	fn	sensitivity_with_ci	specificity_with_ci	precision_with_ci	accuracy_with_ci	f1_point_only
10	5	0	22	0	3	0	100.0% (95% CI 85.1%-100.0%)	0.0% (95% CI 0.0%-56.1%)	88.0% (95% CI 70.0%-95.8%)	88.0% (95% CI 70.0%-95.8%)	93.6%
10	10	0	22	0	3	0	100.0% (95% CI 85.1%-100.0%)	0.0% (95% CI 0.0%-56.1%)	88.0% (95% CI 70.0%-95.8%)	88.0% (95% CI 70.0%-95.8%)	93.6%
10	20	0.3	22	1	2	0	100.0% (95% CI 85.1%-100.0%)	33.3% (95% CI 6.1%-79.2%)	91.7% (95% CI 74.2%-97.7%)	92.0% (95% CI 75.0%-97.8%)	95.7%
10	30	0.35	22	1	2	0	100.0% (95% CI 85.1%-100.0%)	33.3% (95% CI 6.1%-79.2%)	91.7% (95% CI 74.2%-97.7%)	92.0% (95% CI 75.0%-97.8%)	95.7%

angle_deg	thickness_vx	selected_threshold	tp	tn	fp	fn	sensitivity_with_ci	specificity_with_ci	precision_with_ci	accuracy_with_ci	f1_point_only
10	50	0.25	22	2	1	0	100.0% (95% CI 85.1%-100.0%)	66.7% (95% CI 20.8%-93.9%)	95.7% (95% CI 79.0%-99.2%)	96.0% (95% CI 80.5%-99.3%)	97.8%
10	100	0.15	22	1	2	0	100.0% (95% CI 85.1%-100.0%)	33.3% (95% CI 6.1%-79.2%)	91.7% (95% CI 74.2%-97.7%)	92.0% (95% CI 75.0%-97.8%)	95.7%
15	5	0	22	0	3	0	100.0% (95% CI 85.1%-100.0%)	0.0% (95% CI 0.0%-56.1%)	88.0% (95% CI 70.0%-95.8%)	88.0% (95% CI 70.0%-95.8%)	93.6%
15	10	0	22	0	3	0	100.0% (95% CI 85.1%-100.0%)	0.0% (95% CI 0.0%-56.1%)	88.0% (95% CI 70.0%-95.8%)	88.0% (95% CI 70.0%-95.8%)	93.6%
15	20	0	22	0	3	0	100.0% (95% CI 85.1%-	0.0% (95% CI 0.0%-56.1%)	88.0% (95% CI 70.0%-	88.0% (95% CI 70.0%-	93.6%

angle_deg	thickness_vx	selected_threshold	tp	tn	fp	fn	sensitivity_with_ci	specificity_with_ci	precision_with_ci	accuracy_with_ci	f1_point_only
							100.0%)		95.8%)	95.8%)	
15	30	0	22	0	3	0	100.0% (95% CI 85.1%-100.0%)	0.0% (95% CI 0.0%-56.1%)	88.0% (95% CI 70.0%-95.8%)	88.0% (95% CI 70.0%-95.8%)	93.6%
15	50	0.25	22	2	1	0	100.0% (95% CI 85.1%-100.0%)	66.7% (95% CI 20.8%-93.9%)	95.7% (95% CI 79.0%-99.2%)	96.0% (95% CI 80.5%-99.3%)	97.8%
15	100	0	22	1	2	0	100.0% (95% CI 85.1%-100.0%)	33.3% (95% CI 6.1%-79.2%)	91.7% (95% CI 74.2%-97.7%)	92.0% (95% CI 75.0%-97.8%)	95.7%
30	5	0	22	0	3	0	100.0% (95% CI 85.1%-100.0%)	0.0% (95% CI 0.0%-56.1%)	88.0% (95% CI 70.0%-95.8%)	88.0% (95% CI 70.0%-95.8%)	93.6%
30	10	0	22	0	3	0	100.0% (95% CI	0.0% (95% CI 0.0%	88.0% (95% CI	88.0% (95% CI	93.6%

angle_deg	thickness_vx	selected_threshold	tp	tn	fp	fn	sensitivity_with_ci	specificity_with_ci	precision_with_ci	accuracy_with_ci	f1_point_only
							85.1%-100.0%)	-56.1%)	70.0%-95.8%)	70.0%-95.8%)	
30	20	0	22	0	3	0	100.0% (95% CI 85.1%-100.0%)	0.0% (95% CI 0.0%-56.1%)	88.0% (95% CI 70.0%-95.8%)	88.0% (95% CI 70.0%-95.8%)	93.6%
30	30	0	22	0	3	0	100.0% (95% CI 85.1%-100.0%)	0.0% (95% CI 0.0%-56.1%)	88.0% (95% CI 70.0%-95.8%)	88.0% (95% CI 70.0%-95.8%)	93.6%
30	50	0	21	1	2	1	95.5% (95% CI 78.2%-99.2%)	33.3% (95% CI 6.1%-79.2%)	91.3% (95% CI 73.2%-97.6%)	88.0% (95% CI 70.0%-95.8%)	93.3%
30	100	0.15	17	2	1	5	77.3% (95% CI 56.6%-89.9%)	66.7% (95% CI 20.8%-93.9%)	94.4% (95% CI 74.2%-99.0%)	76.0% (95% CI 56.6%-88.5%)	85.0%
45	5	0	22	0	3	0	100.0%	0.0% (95%	88.0%	88.0%	93.6%

angle_deg	thickness_vx	selected_threshold	tp	tn	fp	fn	sensitivity_with_ci	specificity_with_ci	precision_with_ci	accuracy_with_ci	f1_point_only
							(95% CI 85.1%-100.0%)	CI 0.0%-56.1%)	(95% CI 70.0%-95.8%)	(95% CI 70.0%-95.8%)	
45	10	0	22	0	3	0	100.0% (95% CI 85.1%-100.0%)	0.0% (95% CI 0.0%-56.1%)	88.0% (95% CI 70.0%-95.8%)	88.0% (95% CI 70.0%-95.8%)	93.6%
45	20	0	22	0	3	0	100.0% (95% CI 85.1%-100.0%)	0.0% (95% CI 0.0%-56.1%)	88.0% (95% CI 70.0%-95.8%)	88.0% (95% CI 70.0%-95.8%)	93.6%
45	30	0	22	0	3	0	100.0% (95% CI 85.1%-100.0%)	0.0% (95% CI 0.0%-56.1%)	88.0% (95% CI 70.0%-95.8%)	88.0% (95% CI 70.0%-95.8%)	93.6%
45	50	0.2	19	2	1	3	86.4% (95% CI 66.7%-95.3%)	66.7% (95% CI 20.8%-93.9%)	95.0% (95% CI 76.4%-99.1%)	84.0% (95% CI 65.3%-93.6%)	90.5%

angle_deg	thickness_vx	selected_threshold	tp	tn	fp	fn	sensitivity_with_ci	specificity_with_ci	precision_with_ci	accuracy_with_ci	f1_point_only
45	100	0	15	2	1	7	68.2% (95% CI 47.3%-83.6%)	66.7% (95% CI 20.8%-93.9%)	93.8% (95% CI 71.7%-98.9%)	68.0% (95% CI 48.4%-82.8%)	78.9%
