# Supplementary material for: Zero-Shot Domain Adaptation of 2D Mammography Lesion Detection Model for 3D Breast CT Malignancy Detection
Source: Cancers (Basel). 2026 Aug 11;18(16):2585. doi: 10.3390/cancers18162585 (PMC13511489; doi:10.3390/cancers18162585)
Supplement: Supplementary file 1 [file cancers-18-02585-s001.zip › Supplementary Table S4_ 3D image-level Benign vs. Malignant Classification AUC.pdf]

Supplementary Table S4: 3D image-level Benign vs. Malignant Classification AUC

Angle (°)	Thickness (vx)	Classification AUC	95% CI
30	5	0.7197	0.0000–0.8406
45	30	0.7121	0.0000–0.8182
15	20	0.6970	0.0000–0.8333
15	10	0.6894	0.0000–0.8188
15	30	0.6742	0.0000–0.8188
10	30	0.6667	0.0000–0.8175
30	30	0.6591	0.0000–0.7976
10	50	0.6515	0.0000–0.7121
15	50	0.6515	0.0000–0.7143
10	10	0.6212	0.0000–0.7386
30	20	0.5985	0.0000–0.7302
10	20	0.5758	0.0000–0.7143
30	10	0.5455	0.0000–0.7053
45	5	0.5379	0.0000–0.7174
45	20	0.5379	0.0000–0.7101
15	5	0.5227	0.0000–0.7063
10	100	0.4848	0.0000–0.6449
45	10	0.4848	0.0000–0.5952
10	5	0.4697	0.0000–0.6667
45	50	0.4167	0.0000–0.6848
15	100	0.3788	0.0000–0.6023
30	50	0.3182	0.0000–0.5076

Angle (°)	Thickness (vx)	Classification AUC	95% CI
30	100	0.3106	0.0000–0.5217
45	100	0.1136	0.0000–0.3600
