# Supplementary material for: Zero-Shot Domain Adaptation of 2D Mammography Lesion Detection Model for 3D Breast CT Malignancy Detection
Source: Cancers (Basel). 2026 Aug 11;18(16):2585. doi: 10.3390/cancers18162585 (PMC13511489; doi:10.3390/cancers18162585)
Supplement: Supplementary file 1 [file cancers-18-02585-s001.zip › Supplementary Table S5_ 2D slab-projection object-detection performance.pdf]

Supplementary Table S5: 2D slab-projection object-detection performance

Angle(°)	Thickness(vx)	Best-F1 threshold	Object precision	Object recall	Object F1	AP@IoU 0.3	AP@IoU 0.5
10	5	0.3	33.8% (95% CI 33.2–34.3%)	14.7% (95% CI 14.4–15.1%)	20.5% (95% CI 20.1–20.9%)	9.7% (95% CI 9.4–10.0%)	4.7% (95% CI 4.5–4.9%)
10	10	0.2	32.5% (95% CI 31.6–33.3%)	15.4% (95% CI 14.8–15.9%)	20.9% (95% CI 20.2–21.5%)	9.8% (95% CI 9.3–10.3%)	4.7% (95% CI 4.4–5.1%)
10	20	0.1	27.2% (95% CI 26.2–28.1%)	15.9% (95% CI 15.2–16.7%)	20.1% (95% CI 19.3–20.9%)	8.9% (95% CI 8.3–9.5%)	3.6% (95% CI 3.3–4.1%)
10	30	0.05	23.9% (95% CI 22.9–24.9%)	16.6% (95% CI 15.7–17.6%)	19.6% (95% CI 18.6–20.5%)	8.1% (95% CI 7.4–8.8%)	3.0% (95% CI 2.6–3.5%)
10	50	0	23.9% (95% CI 22.5–25.2%)	15.0% (95% CI 13.9–16.1%)	18.4% (95% CI 17.3–19.5%)	6.9% (95% CI 6.1–7.8%)	2.4% (95% CI 2.0–2.9%)
10	100	0.05	26.8% (95% CI 24.4–29.2%)	9.3% (95% CI 8.3–10.5%)	13.9% (95% CI 12.4–15.4%)	4.4% (95% CI 3.7–5.3%)	1.7% (95% CI 1.2–2.2%)
15	5	0.25	30.9% (95% CI 30.3–31.6%)	15.3% (95% CI 14.9–15.7%)	20.5% (95% CI 20.0–21.0%)	9.7% (95% CI 9.3–10.1%)	4.7% (95% CI 4.4–5.0%)
15	10	0.2	32.8% (95% CI	15.5% (95% CI	21.1% (95% CI	9.8% (95% CI	4.7% (95% CI

Angle(°)	Thickness(vx)	Best-F1 threshold	Object precision	Object recall	Object F1	AP@IoU 0.3	AP@IoU 0.5
			31.8–33.8%)	14.9–16.2%)	20.4–21.8%)	9.3–10.5%)	4.3–5.1%)
15	20	0.1	27.3% (95% CI 26.2–28.6%)	16.0% (95% CI 15.0–17.0%)	20.2% (95% CI 19.2–21.2%)	8.9% (95% CI 8.2–9.7%)	3.7% (95% CI 3.2–4.2%)
15	30	0.05	23.7% (95% CI 22.5–25.0%)	16.7% (95% CI 15.5–17.9%)	19.6% (95% CI 18.5–20.7%)	8.2% (95% CI 7.4–9.2%)	3.1% (95% CI 2.7–3.7%)
15	50	0	24.1% (95% CI 22.5–25.7%)	14.9% (95% CI 13.6–16.3%)	18.4% (95% CI 17.1–19.8%)	7.1% (95% CI 6.2–8.2%)	2.5% (95% CI 2.0–3.2%)
15	100	0	25.1% (95% CI 22.6–27.8%)	9.9% (95% CI 8.6–11.4%)	14.2% (95% CI 12.5–16.0%)	4.6% (95% CI 3.7–5.8%)	1.7% (95% CI 1.2–2.4%)
30	5	0.2	28.2% (95% CI 27.4–29.0%)	16.4% (95% CI 15.8–17.1%)	20.7% (95% CI 20.1–21.4%)	10.0% (95% CI 9.5–10.6%)	4.8% (95% CI 4.4–5.2%)
30	10	0.2	33.0% (95% CI 31.6–34.4%)	15.9% (95% CI 14.9–16.9%)	21.5% (95% CI 20.4–22.6%)	10.3% (95% CI 9.5–11.2%)	4.8% (95% CI 4.3–5.5%)
30	20	0.15	31.4% (95% CI 29.4–33.3%)	14.9% (95% CI 13.6–16.3%)	20.2% (95% CI 18.7–21.7%)	9.1% (95% CI 8.1–10.3%)	3.8% (95% CI 3.1–4.6%)
30	30	0.05	24.0% (95% CI 22.2–25.7%)	17.2% (95% CI 15.5–18.9%)	20.0% (95% CI 18.4–21.6%)	8.6% (95% CI 7.4–10.0%)	3.3% (95% CI 2.6–4.1%)
30	50	0	24.6% (95% CI	15.4% (95% CI	19.0% (95% CI	7.2% (95% CI	2.6% (95% CI

Angle(°)	Thickness(vx)	Best-F1 threshold	Object precision	Object recall	Object F1	AP@IoU 0.3	AP@IoU 0.5
			22.2–27.0%)	13.6–17.4%)	17.0–20.9%)	5.9–8.8%)	1.8–3.6%)
30	100	0.05	26.1% (95% CI 22.2–29.9%)	9.4% (95% CI 7.7–11.3%)	13.8% (95% CI 11.5–16.3%)	4.5% (95% CI 3.3–6.1%)	1.7% (95% CI 1.1–2.7%)
45	5	0.25	30.9% (95% CI 29.8–32.0%)	16.4% (95% CI 15.6–17.3%)	21.4% (95% CI 20.6–22.3%)	10.4% (95% CI 9.7–11.1%)	4.9% (95% CI 4.5–5.4%)
45	10	0.25	36.0% (95% CI 34.2–38.0%)	15.8% (95% CI 14.6–17.1%)	22.0% (95% CI 20.6–23.5%)	10.5% (95% CI 9.5–11.7%)	4.8% (95% CI 4.2–5.6%)
45	20	0.1	28.2% (95% CI 26.2–30.3%)	17.5% (95% CI 15.7–19.4%)	21.6% (95% CI 19.8–23.4%)	9.5% (95% CI 8.2–11.1%)	4.0% (95% CI 3.2–5.0%)
45	30	0	22.4% (95% CI 20.4–24.4%)	18.1% (95% CI 16.0–20.4%)	20.0% (95% CI 18.1–22.0%)	8.6% (95% CI 7.2–10.4%)	3.3% (95% CI 2.5–4.4%)
45	50	0	22.9% (95% CI 20.2–25.6%)	15.3% (95% CI 13.0–18.0%)	18.4% (95% CI 16.0–20.9%)	7.0% (95% CI 5.5–9.1%)	2.6% (95% CI 1.8–4.0%)
45	100	0	23.6% (95% CI 19.3–28.0%)	10.1% (95% CI 7.8–12.9%)	14.2% (95% CI 11.3–17.5%)	4.5% (95% CI 3.1–6.7%)	1.6% (95% CI 0.8–2.9%)
